# Identification of capacity development indicators for faculty development programs: A nominal group technique study

**DOI:** 10.1186/s12909-020-02068-7

**Published:** 2020-05-24

**Authors:** Mahla Salajegheh, Roghayeh Gandomkar, Azim Mirzazadeh, John Sandars

**Affiliations:** 1 Department of Medical Education, School of Medicine, Tehran University of Medical Sciences, No. 57, Hojjatdust Alley, Naderi St., Keshavarz Blvd, Tehran, 141663591 Iran; 2 Education Development Center, Tehran University of Medical Sciences, Tehran, Iran; 3 Health Professions Education Research Center, Tehran University of Medical Sciences, Tehran, Iran; 4 Department of Internal Medicine, School of Medicine, Tehran University of Medical Sciences, Tehran, Iran; 5 Edge Hill University Medical School, Edge Hill University, Ormskirk, UK

**Keywords:** faculty development, capacity development, indicator, nominal group technique

## Abstract

**Background:**

Although there have been many research studies of the effectiveness of faculty development in health profession education, the contribution of these programs to organizational development through capacity development has not been studied. Further understanding of capacity development requires appropriate indicators and no previous indicators for faculty development of health profession educators were identified. The aim of the study was to identify indicators of capacity development in the context of faculty development programs at Tehran University of medical sciences (TUMS).

**Methods:**

A nominal group technique session was conducted with key informants from faculty development program providers to generate and prioritize a list of capacity development indicators.

**Results:**

A list of 26 indicators was generated and five categories were identified: Development and innovation in teaching and learning process, Development and innovation in communication and collaboration at different levels, Development and sustaining faculty development programs, Development of educational leadership and management, Development in scholarship.

**Conclusions:**

Capacity development for faculty development interventions of health profession educators is a process of engagement within a wider system, including individual and collective action, and involves the socialization of the teachers into suitable roles through professional identity development and participation within the wider system.

## Background

Changing concepts of education and the increasing complexity of healthcare have led many medical universities to design and implement a variety of faculty development programs in order to help health profession educators to effectively perform their multiple roles which include teaching, leadership and production of resources [1]. Faculty development involves all of the activities that are designed to help health profession educators to improve theknowledge, skills and behaviors that are required for their different roles, both as an individual and within organizational settings [2].

Faculty development is essential for any medical university, with the development of a basic collection of competencies in faculty members that enable them to cope with their workload and environment changes so that they can adequately perform their critical functions [3]. Faculty development can also be a useful approach for the advancement of organizational development by producing a sense of support, inspiration and energy, carrying out change and creativity, improving organizational capabilities, and creating a future workforce of skilled educators [4, 5].

In spite of the potential contribution of faculty development programs to organizational development, studies tend to focus on only measuring the outcomes at an individual level. Most studies have focused on the evaluation of participant satisfaction immediately after they attended a program [6–8]. Some studies have explored learning, which included attitudinal changes and increases in knowledge or skills [9–11]. A few studies have assessed the changes in educational behavior of the participants in their multiple roles [12, 13]. Most of these evaluations have also only had a focus on short term outcomes, especially at an individual level.

Policy makers and funding agencies are increasing their demands for evaluating change at a much broader level, seeking evidence of impact for potential sustainability of the faculty development programs by wider involvement of the participants in their sphere of work and at the organizational level [14]. However, faculty development programs occur within rapidly changing organizations in which people work and the impact of these programs is not linear [15]. The simple linear program evaluation models consider that the acquisition of new knowledge and skills will produce organizational change but these models usually ignore the context, with its complexity of factors that influence the outcomes [16, 17].

The concept of capacity development has been expanded to recognize the importance of development as a system-wide process and has increasingly been used to inform an understanding of the complexity of how the organizational impact of human development programs can be achieved [18, 19]. Capacity development has a focus on how changes in human behavior, such as the growth of new attitudes, values, knowledge, skills, and relations hips with others, are generated over time within a complex organizational system [20]. This generative process leads to the enhanced capability of organizations to perform within the complex environment. An essential feature of this process is that the development of individuals become embedded in a collective manner through their relationships with others [20]. A recent study of a faculty development program in South Africa appears to be the first to demonstrate the important features of the capacity development process in health profession education, with collaborative knowledge sharing and support across to produce individual personal and professional development but also change in the organizations in which they worked [21]. Capacity development is essential for the sustainability of faculty development programs, with the evolution in the professional identity and empowerment of faculty members to become change agents who can shape a wider impact in their workplace [17]. The professional identity development occurs at both individual and collective levels through the relationships with others in the workplace and the broader context of the workplace [22–24]. This collective ability through wider sharing is a key characteristic of capacity development and enables the organization to effectively cope with the complexity of environmental changes [2, 25]. Evaluation of the broader capacity development impact of faculty development programs for health profession education requires indicators that can be used to inform evaluations. No previous published research about the indicators of capacity development of faculty development programs for health profession educators were identified.

The aim of this study is to identify the indicators of capacity development in the context of faculty development programs at Tehran University of medical sciences (TUMS). The TUMS Education Development Centre introduced a comprehensive faculty development program in 2015 with the aim of improving the quality of teaching and learning, to engage individuals in collective education development actions, and to promote student learning. A variety of faculty development activities were designed and implemented to address the needs of health profession educators throughout the university. These activities were in different formats, including workshops and seminars, short courses and fellowships, and covered a variety of topics in medical education. One of these programs, the “Basic Teaching Skills Course”, covers essential subjects for teaching effectiveness such as instructional design, teaching methods and, student assessment. This is a longitudinal course designed for new or less experienced faculty members. Participating in this course is essential for all assistant professors for promotion to associate professor. It is delivered in an interactive format with lectures, group work, and assignments and feedbacks. Faculty members from all eleven schools of TUMS are the participants in this program. We chose to study the “Basic Teaching Skills Course” since it has a large number of participants and covers the basic elements of teaching and learning. Hence, we expected that networking and both individual and collective development would occur.

## Method

We used nominal group technique (NGT) to elicit the indicators of capacity development for health profession education in the context of the “Basic Teaching Skills Course” at TUMS in December, 2017. The NGT method was chosen because it promotes the active participation of all group members to produce new ideas through a brain storming format. We followed a classic NGT technique including 1) silent generation of responses to a particular question, 2) round-robin sharing and recording of ideas, 3) group discussion for clarification, and 4) voting on items, with respect to importance, or relatedness [26].

Nineteen key informants were invited via email and, then invitations were confirmed in person. The informants were recruited based on their long-standing association with the TUMS Education Development Center and all had previous experience in the management and administration of faculty development programs, some in the “Basic Teaching Skills Course”. The TUMS’s institutional review board approved the study, participants did not receive any incentives, and participation was voluntary. Verbal consent for participation was obtained based on the proposal approved by the ethics committee.

The NGT meeting was facilitated and lasted 3 hours. In the first step of the NGT, we gave participants a brief description of the NGT process, details of the capacity development concept as a process and the need to have capacity development indicators. We clarified that the indicators could identify the growth and development of attitudes, values, behaviors, abilities, motivations, and identity, and could also help to identify changes in persons, and organizations, as they try to develop their performance. We also presented some examples which would not be appropriate as capacity development indicators, such as an increase in the number of trained faculty members, increase in the number of publications, or increase in adopting educational leadership roles. We then provided participants with some examples of capacity development indicators that have been used to evaluate personnel development in various settings such as energy and environmental programs, welfare programs and community management. The aim of providing examples was to clarify an abstract concept such as capacity development. These examples were only provided as a short initial Power Point presentation, with the intention to raise awareness and to avoid participants simply replicating the examples.

Participants were asked the following questions: “what are the indicators of capacity development in the context of the Basic Teaching Skills Course?” and “how may participants in Basic Teaching Skills Course contribute to organizational development (in education)?” and we asked each participant to independently and privately write down individual notes what he or she considered as the indicators of capacity development of the “Basic Teaching Skills Course”. During this phase of the NGT process, the participants worked alone and were not permitted to talk to others in the room or to ask for clarification of the questions. The indicators were then shared with the group in a round-robin format, with each member sharing one item from their list, without naming any previously mentioned items, until all items had been exhausted. All the named indictors were typed by a facilitator in full view of all the others. During this phase of the process, no questions were allowed.

In the next phase, with the assistance of the facilitator, participants discussed the list of indicators, resolved questions of clarity and combined related indicators into a single item and clarification was provided. In cases of dispute, the person who raised the indicator could decide whether or not to combine it with another item. Similar indicators were grouped through group discussion and a label was selected for each category. During the next phase, we followed the nominal group technique and asked participants to vote privately on the indicators and rank the indicators on a Likert scale, where 1 was “completely not related” to capacity development of faculty development and 5 was “completely related”.

Finally, voting results were summed across participants and mean score of votes for each indicator was calculated to determine the most related indicators of capacity development of faculty development programs for health profession educators. For this study, we only determined the related indicators with a mean score greater than or equal to 3.50.

## Results

Nine key informants agreed to participate in the NGT. The majority of them (66.7%) were women. Almost half (44.4%) were assistant professors, 33.3% were associate professors, 11.1% was professor and the rest were instructors.

The initial list of capacity development indicators of the “Basic Teaching Skills Course” consisted of 88 items organized in six categories. After the voting step, the list of indicators was reduced to 26 indicators in five categories. These five categories were: Development and innovation in teaching and learning process, Development and innovation in communication and collaborations at different levels, Development and sustaining faculty development programs, Development of educational leadership and management, Development in scholarship.

The development and innovation in teaching and learning process category has a focus on developing competencies in the teaching and learning process, including various teaching and student assessment methods. Development and innovation in communication and collaboration at different levels includes networking and the relationships within the community of medical colleagues. The category of development and sustaining faculty development programs represents the interest of teachers in medical education and their support and collaboration with colleagues, which is essential to sustain and develop the programs. Development of educational leadership and management category refers to involvement in the development, implementation and evaluation of the medical education institution. Development in scholarship represents educational research and scholarship, including involvement in activities that share new knowledge. For descriptive purposes, the capacity development indicators of faculty development programs are presented within their assigned category in Table 1.

**Table 1.** Capacity development indicators of faculty development programs

Category 1: Development and innovation in teaching and learning process	Mean score of each indicator
Enhanced competencies to transfer concepts and skills to learners	4.83
Improved competencies to manage the classroom	4.83
Competencies to apply novel methods for assessing learners	4.83
Competencies to design a course plan based on educational design principles	4.67
Competencies to provide feedback to learners	4.67
Motivation to receive feedback on their own teaching performance	4.50
Meet the principles of professional behavior in education and clinical practice	4.50
Competencies to motivate students for learning	4.34
Competencies to apply interactive teaching methods aligned with educational conditions	4.34
Ensure fairness in teaching and assessment of learners	4.16
Enhanced enthusiasm and self-confidence in teaching	3.52
Improved teaching quality	3.52
**Category 2: Development and innovation in communication and collaboration at different levels**	**Mean score of each indicator**
Competencies to communicate with learners, colleagues and patients appropriately	4.50
Help new colleagues for career progression	3.67
Competencies to do teamwork	3.67
**Category 3: Development and sustaining faculty development programs**	**Mean score of each indicator**
Refer to specialized evidence or consult with experts when answering a question or to inform decision making in the field of medical education	4.33
Efforts to be up-to-date in the field of medical education	3.86
More motivation to become familiar with various fields of medical education	3.69
More motivation for participating in new faculty development programs	3.69
Encourage and provide guidance to other colleagues to participate in faculty development programs	3.69
**Category 4: Development of educational leadership and management**	**Mean score of each indicator**
Cooperate in the implementation of educational development processes at university/school	4.50
Motivation to analysis the university/school policies regarding educational activities	4.16
Motivation to evaluate the quality of education in their own department	4.16
**Category 5: Development in scholarship**	**Mean score of each indicator**
Motivation to identify educational problems, and, to design and implement appropriate interventions	4.50
Motivation to attend seminars and conferences related to medical education	4.00
Competencies to use medical education evidence in my educational activities	3.52

The most important capacity development indicators that were identified were within the development and innovation in teaching and learning process category, including enhanced competencies to transfer concepts and skills to learners, improved competencies to manage the classroom and competencies to apply novel methods for assessing learners (mean = 4.83). In the development and innovation in communication and collaboration at different levels category, the most important indicator of capacity development was competencies to communicate with learners, colleagues and patients appropriately (mean = 4.50). The highest score for a capacity development indicator in the development and sustaining faculty development programs category was refer to specialized evidence or consult with experts when answering a question or to inform decision making in the field of medical education (mean = 4.33). In the development of educational leadership and management category, the main capacity development indicator was to cooperate in the implementation of educational development processes at university/school (mean = 4.50). The most important capacity development indicator for development in scholarship category included motivation to identify educational problems, and to design and implement appropriate interventions (mean = 4.5).

## Discussion

Our study is the first, to our knowledge, to identify the indicators of capacity development for faculty development programs in health profession education. The study identified five categories of capacity development indicators that were considered by key faculty members to be important outcomes for the “Basic Teaching Skills Course” at TUMS.

We found that the “Development and innovation in teaching and learning process” was one of the main categories of capacity development indicators for faculty development programs. This is consistent with the aims and expected learning outcomes of most faculty development programs. An important outcome of these programs is that participants begin to thoughtfully apply their new knowledge and skills to modify their teaching by increasing their use of a variety of educational methods to influence curriculum development, including course design and organization [27]. Evans et al (2013) described and reflected on their experience of running a 2 years nursing faculty development program and noted that developing innovations in teaching and learning was one of the main outcomes [28]. In our study, enhanced competencies to transfer concepts and skills to learners were prioritized highly, which is supported by the importance of the ‘information provider’ role of medical teachers [29]. Because of the importance of this role, most faculty development programs aim to help teachers to become more effective in this role [8, 30–33]. The importance of improved competencies to manage the classroom has been previously noted, [34]. One of the aims of faculty development programs, especially new instructors, is to help them to find solutions to their classroom problems and experiment management processes consistent with their own education values and those of their employing universities [35, 36].

The “development and innovation in communication and collaboration at different levels” was identified as another important category representative of capacity development of our faculty development programs. This finding has been noted in two previous studies that had a focus on capacity development. Frantz et al (2015) investigated the perceptions of the personal and professional impact of the participants of a faculty development program in sub-Saharan Africa [37]. Participants reported that the program had given them the opportunity and skills to communicate and engage with other colleagues and education interest groups. Another study by Frantz et al (2019) investigated the contribution of a faculty development program to individual and collective capacity development in Africa. One of their emergent themes was networking which was identified as providing both critical personal support and engagement within a wider professional community of practice [21]. Despite the importance of this category, two of the identified indicators (“help new colleague for career progression" and "competences to do teamwork") had low scores. This may be because of cultural barriers, lack of appropriate opportunities for communication, and conflict of interests [38]. Previous studies have noted that teamwork and networking is less valued in some developing countries [39]. To fulfill TUMS comprehensive program, faculty development programs that foster the creation of mutual and common values, beliefs and norms among the participants within a safe and supportive environment encourage cooperation and networking [40].

In the “Development and sustaining faculty development programs” category most of the capacity development indicators that were identified are consistent with the impact of faculty development programs which have been reported by prior studies. However, we found some new and important indicators in relation to the increased motivation and effort of participants of these programs to become familiar with medical education. This finding highlights the high commitment that is demonstrated by many participants on faculty development programs for health profession education.

The “cooperate in the implementation of educational development processes at university/school” category, is identified as being an important indicator in the “Development of educational leadership and management” category. This finding emphasizes the importance of preparing health profession educators for educational leadership roles and responsibilities [41]. Our findings are consistent with Schreurs et al (2016) work that reported that faculty development programs increased understanding of educational organizations and their policies and also enhanced confidence to implement educational development process, which generated improvement in the educational culture of the organization [42]. Participants also considered that “Development in scholarship” was an important category, with motivation to identify educational problems, and to design and implement appropriate interventions. Frantz et al (2019) identified educational research and scholarship among their themes about the contribution of a faculty development program to individual and collective capacity development in Africa. They reported that the development of the participants’ individual knowledge and skills in research and scholarship led to the individual and collective advancement of scholarly educational activities [21].

An interesting finding of our study is the high agreement about the importance of collective agency of the teachers in the organization in which they work, with the faculty development program enhancing the motivation and confidence of participants to take collective action for organizational change. This capacity development outcome requires active engagement in the wider sharing of acquired competencies and the co-development of capabilities at a system level. Our findings are consistent with the main aspects of capacity development, which highlights the collective process of interactions in a wider system to address problems and bring about transformational change within a specific context [21]. The development of agency is closely aligned with the evolution of professional identity as a health profession educator and their empowerment to produce organizational change in complex environment [3].

The findings of our study are important, they highlight that the identified indicators of capacity development in the context of faculty development programs for health profession education at TUMS consider not only the impact on individuals but also within the wider TUMS organization. The need for a broader approach to program evaluation that is beyond individual perceptions, acquisition of new learning and changes in behavior is required for future evaluation of faculty development programs. Also, a systems level perspective is essential to expand future faculty development curricula from only teaching competencies to demonstrating communication, leadership and management skills with the purpose of strengthening teachers’ capacity to become organizational change agents [2].

Producing a list of indicators can be a vital part of the capacity development process itself. The initial identification and further refinement of the indicators, such as by the use of NGT, is a collective process and both the process and outcome of producing the indicators can lead to organizational change [20]. An important aspect of the collective process is that a shared consensus about the future direction of a faculty development program can be achieved, which is essential for defining clear objectives of the program, determines the way of monitoring and evaluation and providing resources to support a program [19].

Some limitations of this research should be considered. The study was performed in one organization with a limited number of key informants and represents the indicators of capacity development of only one type of faculty development program; therefore, findings may not be generalizable to other context. However, recruiting participants at a variety stages of profession created a rational list of indicators related to capacity development of faculty development programs for our institution. Further research done at other universities is recommended to determine if there are other significant indicators missed in this pilot study and if these findings generalize over one institution. Collaborative research across universities could lead to the refinement of a list of common indicators that could be used to evaluate the capacity development of faculty development programs for health profession education. The participants were also provided with examples of capacity development indicators before they started to generate indicators, which may have influenced the participants’ answering options. Finally, within the existing relatively limited literature on capacity development in the context of faculty development, there is a noticeable paucity of this type of research in the health profession education context and future research is recommended to understand the process of capacity development.

## Conclusions

Identification of the indicators of capacity development in the context of faculty development programs for health profession education provides insight into the impact of these programs on organizational development. The findings highlight the importance of understanding, implementing and evaluating faculty development programs from a capacity development perspective that considers both the development of individual and collective abilities to positively contribute to change in the wider health profession education system.

## Data Availability

The datasets used and/or analyzed during the current study are available from the corresponding author on reasonable request.

## Abbreviations

TUMS: Tehran University of Medical Sciences
NGT: Nominal Group Technique
